# Rotationplasty in the Elderly

**DOI:** 10.1155/2008/402378

**Published:** 2008-07-29

**Authors:** Jendrik Hardes, Gerhard Ulrich Exner, Dieter Rosenbaum, Arne Streitbuerger, Carsten Gebert, Georg Gosheger, Helmut Ahrens

**Affiliations:** ^1^Department of Orthopaedics, Westfälische Wilhelms University Münster, Albert Schweitzer strasse 33, 48149 Münster, Germany; ^2^Balgrist Hospital, University of Zurich, 8006 Zurich, Switzerland; ^3^Movement Analysis Laboratory, Department of Orthopaedics, Westfälische Wilhelms University Münster, Domagkstrasse 3, 48129 Münster, Germany

## Abstract

*Purpose*. Rotationplasty has proven its efficacy in the treatment of malignant bone tumors of the lower extremity in predominantly young patients. To our knowledge this procedure has not been reported in patients over 60 years before. *Materials and Methods*. 3 patients over 60 years with an A1-rotationplasty because of a sarcoma were included in this study. Complications and functional results were recorded. In one patient an electromyography was done. *Results*. Despite electromyography showing good adaptation of the muscles to the altered function, the functional results of these three patients were limited. two out of three patients needed a cane for walking distances over 200 meters. No secondary amputation was necessary. *Discussion*. Our study demonstrates that rotationplasty is an alternative to an above-knee amputation in older patients but with poorer functional results in comparison to younger patients. However, limb-salvage surgery should be preferred whenever possible.

## 1. INTRODUCTION

Before the era of endoprosthetic devices, rotationplasty, which was introduced in 1974 by Salzer et al. [1] in the surgery of malignant bone tumors and modified by Winkelmann [2, 3] was a viable alternative to amputation. Despite many authors favouring limb saving procedures today, rotationplasty obtains excellent functional and psychosocial results [4, 5]. Whereas nowadays in most patients limb-salvage procedures are performed successfully, rotationplasty can be recommended in tumors with an extensive soft tissue component, as a salvage procedure in the case of a failed limb salvage procedure [5], and in very young children as an alternative to growing prostheses [6].

It is a common question from physicians (especially oncologists), if there is an age limitation for rotationplasty. We know that even very young children can be treated with a rotationplasty with excellent functional results [2, 3, 6], but an upper age limitation is difficult to determine. It depends on the biological age of the patient, the range of motion in the ankle joint, the stage of tumor disease, and the extension of the tumor. To our knowledge there are no reports about rotationplasty in older patients. The current-case report describes the perioperative complications and the short-term followup in two patients and a long-term functional result in one patient with an A1-rotationplasty.

## 2. MATERIALS AND METHODS

**Figure 1:**
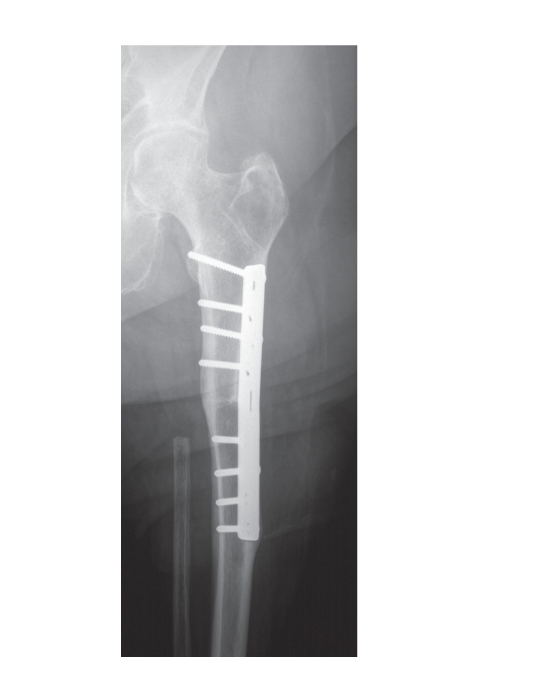
An anterior-posterior radiograph showing an A1-rotationplasty 12 years postoperatively without signs of pseudarthrosis.

**Figure 2:**
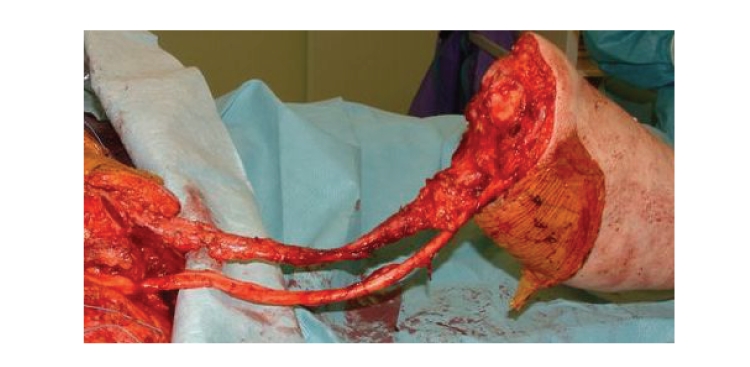
An intraoperative picture after resection of a dedifferentiated liposarcoma of the distal part of the thigh. The femoral vessels could be preserved because of no tumor infiltration. See below the vessels the sciatic nerve.

**Figure 3:**
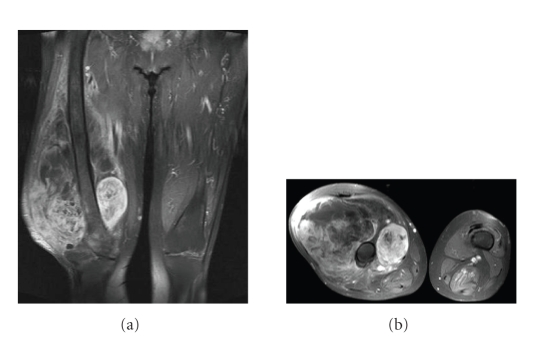
(a) A coronar magnetic resonance imaging (T2-weighted) showing the tumor infiltration of the knee. (b) An axial T2-weighted sequence showing the tumor extension of the whole quadriceps muscles.

Three patients without any comorbidities (e.g., diabetes and atherosclerosis) with an age over 60 years (mean 65, range 62–70) were treated with an AI rotationplasty according to Winkelmann [3] instead of an above-knee amputation (Table 1). This type of rotationplasty is applicable for patients with a tumor in the distal part of the femur or thigh. The osteotomy is performed in the proximal third of the femur, distal to the lesser trochanter, and in the proximal part of the tibia, distal to the tibial tuberosity. The foot is rotated 180°, and the tibia is reattached to the proximal femur by a plate osteosynthesis (Figure 1). The femoral vessels can be resected and reanastomosed or can be preserved in the case of absent tumor infiltration (Figure 2). The indication for rotationplasty was an extensive soft tissue sarcoma of the quadriceps muscle in two patients (1 malignant schwannoma grade III, 1 dedifferentiated liposarcoma). Patient number 1 was treated previously by 3 intralesional resections in other hospitals. Because of local recurrence, a marginal resection combined with adjuvant radiotherapy with 60 Gray was performed in our department. However, 9 months postoperatively a further local recurrence with synchronous lung metastases developed. Patient number 2 with an additional intraarticular tumor extension to the knee had no prior surgery (Figures 3(a) and 3(b)). In these 2 patients, rotationplasty was indicated because of tumor infiltration of the whole quadriceps muscle. Therefore, limb sparing surgery (e.g., with a tumor prosthesis) with good functional results regarding stabilization and extension of the knee was not possible any more. The third patient had a malignant fibrous histiocytoma of the popliteal fossa with an extensive infiltration of the popliteal vessels, which was treated by an intralesional resection one month before in another hospital. The followup ranged from 6 months to 15 years. At final followup, two patients were alive without evidence of disease. Patient number 1 died of disease due to lung metastases 12 months postoperatively.

In this retrospective study the patients charts were evaluated regarding intra- and postoperative complications and the functional results evaluated to Enneking et al. [7]. Data about the functional results were obtained by a questioning. In patient number 3, an electromyography of the involved and uninvolved limb was performed.

## 3. RESULTS

No patient experienced a local recurrence. Bone healing after osteosynthesis of the tibia and proximal femur was achieved in all cases (Figure 1). Regarding postoperative complications, patient number 1 developed a thrombosis of the femoral vein 6 months postoperatively resulting in moderate lymphedema. All patients received full-dose anticoagulation for 1 week and prophylaxis for deep venous thrombosis until the final prosthesis was fitted. No wound healing complications occurred.

**Figure 4:**
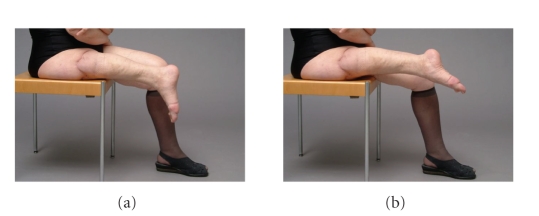
Photographs 6 months postoperatively showing an unrestricted dorsalextension and plantarflexion of the ankle.

**Figure 5:**
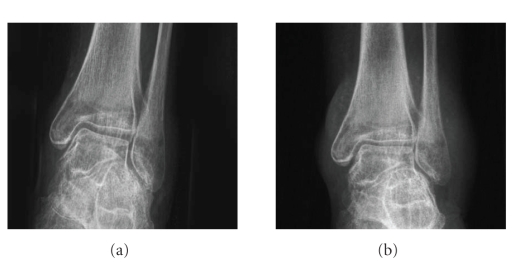
(a) An anterior-posterior radiograph showing the ankle without relevant signs of arthrosis 1 years postoperatively. Osteopenia due to the limited exposure of the leg. (b) An anterior-posterior and lateral radiograph showing the ankle 15 years postoperatively without relevant signs of arthrosis.

Two patients needed a cane for a gait distance of more than 200 meters. The walking distance even with support was reduced but ranged from 500 to 2000 meters. However, no patient experienced pain and needed analgesics. The range of motion of the ankle joint was not restricted (Figure 4). In patient number 2 no signs of degenerative joint disease of the ankle joint could be observed 15 years postoperatively. However, osteopenia due to the limited loading of the rotated leg was obvious (Figures 5(a) and 5(b)). The mean Enneking score was 19 of 30 points. All patients were amenable to having the same surgery again if necessary.

Electromyography in patient number 2 showed that the tibialis anterior muscle converted to a powerful flexor of the ankle joint. The soleus muscle as an extensor of the ankle joint showed normal activity electromyography, but the muscle activity of the lateral gastrocnemius muscle was slightly and of the medial gastrocnemius muscle severely reduced. The peroneal muscles with a stabilizing function of the ankle joint showed only a slightly reduced activity. In summary, electromyography showed despite the reduced activity of the medial gastrocnemius muscle a good adaptation of the muscles to the new function.

## 4. DISCUSSION

Rotationplasty was introduced by Salzer et al. [1] in the surgery of malignant bone tumors of the distal femur as an alternative to an above-knee amputation in young patients. In the following study, Winkelmann [2, 3] widened the indication for rotationplasty for patients with tumors located at the proximal or even entire femur and the proximal tibia. Many studies show the good functional results and suggest good psychosocial acceptance by patients [8, 9]. In contrast to an above-knee amputation, rotationplasty allows the patients to actively control the neo-knee mechanism, which results in a coordinated gait pattern [9]. The muscles of the ankle or neo-knee joint are able to adapt very fast to the changed anatomic condition [9]. Further studies underline that an adaption of the ankle joint to the changed biomechanic situation is possible and degenerative changes of the joint could be excluded in a long-term followup ranging between 10 and 15 years in predominantly young or middle-aged patients [10]. Nevertheless, there have been concerns if this adaptation process is at all possible in older patients. The long-term followup of patient number 2 in the current study could exclude a relevant progressive arthrosis in the ankle joint 15 years postoperatively without having the same activity level as younger patients.

The complication rate of rotationplasty has been reported to be low [2–4]. The worst complications are a failure of the vascular anastomosis and a pseudarthrosis of the osteosynthesis in A1 rotationplasty [2]. To our knowledge rotationplasty has not been reported in patients over 60 years so far. It could be assumed that the complication rate in older patients would be higher, especially regarding wound healing problems and a failure or thrombosis of the vascular anastomosis because of arteriosclerosis. Indeed, a thrombosis of the femoral vein occurred in one out of three patients 6 months postoperatively without relevant clinical complaints. However, thrombosis after rotationplasty has been reported even in younger patients [11]. Furthermore, the perioperative complication rate in older patients with multiple morbidities undergoing an above-knee amputation due to, for instance, arteriosclerosis or diabetes has been reported to be high [12]. Common complications include stump healing problems and cardiovascular morbidities [12, 13]. In our study wound healing complications were not observed. To our knowledge there have been no reports analyzing the complication rate of above-knee amputations due to a sarcoma in older patients.

Regarding the functional results of rotationplasty, Hillmann et al. [14] reported that the age at the time of operation showed a distinct influence on gait, walking ability, and the Enneking score. Younger patients were better able to adapt to the altered anatomical and functional conditions. Our case report could show that an old patient has more difficulties to adapt to the new anatomical situation, in spite of the fact that electromyography showed no marked differences in comparison to younger patients in one patient and the range of motion of the neoknee joint was not limited, the functional results of our patients were poorer (Enneking score of 19 out of 30 points) compared to younger patients reported by Hillmann et al. [14]. They reported an average score of 23.9 out of 30 points in 43 patients with a mean age of 17.8 years. Functional results were poorer in our patients because of a reduced walking distance and by the use of a walking aid for longer distances. However, an above-knee amputation in older patients with comorbidities (diabetes, vascular diseases) is associated with poorer functional results in comparison to younger patients also [12, 15]. Frequently, these patients are not able to walk or do not use their exoprosthesis. Others can walk with the help of crutches for short distances only [12, 15].

It is well known that the energy demand for prosthetic gait is higher in above-knee amputations compared to below-knee amputations [16]. Therefore, rotationplasty could offer a functional improvement in comparison to an above-knee amputation even in older patients because the knee joint is “preserved.” Previous studies showed that patients with a rotationplasty walk more efficiently according to the measurement of consumption of oxygen in comparison to patients with an above-knee amputation [17]. These facts should be transferable as well for older patients, in whom an efficient use of the energy demand is more important because of an age-dependent limitation of the cardiovascular system.

It has to be emphasized that only one patient has a long-term follow-up. Therefore, the results from this patient can not be generalized. The followup of the other two patients is too short to evaluate the functional and radiological results. Furthermore, rotationplasty should not be performed in patients with metastatic disease and a poor prognosis (patient number 1) because of the prolonged rehabilitation process after this surgical procedure. Nevertheless, we believe that this case report is important to show that rotationplasty is a possible procedure even in the elderly. Larger retrospective studies or even prospective studies are needed to examine this topic and to compare patients with rotationplasty against an above-knee amputation.

## 5. CONCLUSION

In conclusion, it can be stated that rotationplasty is an alternative to above-knee amputation in older patients if limb-sparing surgery is not possible. However, the functional results might be not comparable to younger patients, who regain mainly normal walking abilities and sporting abilities. Prospective studies with a larger patient group have to evaluate, if rotationplasty offers a functional advantage against an above-knee amputation in older patients.

## Figures and Tables

**Table 1:** Patient's details.

No.	Gender	Age at operation (years)	Diagnosis	Site of tumor	Metas-tasis	Prior operations	Radio-therapy	Chemo- therapy	Followup (months)	Stage of disease
1	male	62	Malignant schwannoma GII-III	Ventral thigh	Lung	Intralesional resection	Yes	No	12	DOD
2	female	70	Liposarcoma GIII	Ventral thigh	No	No	No	No	6	NED
3	female	63	Malignant fibrous histiocytoma GIII	Popliteal fossa	No	Intralesional resection	No	Yes	186	NED

DOD: Dead of disease, NED: No evidence of disease.
